# Natural phytochemicals that affect autophagy in the treatment of oral diseases and infections: A review

**DOI:** 10.3389/fphar.2022.970596

**Published:** 2022-08-25

**Authors:** Xi Cheng, Qianming Chen, Ping Sun

**Affiliations:** Stomatology Hospital, School of Stomatology, Zhejiang University School of Medicine, Clinical Research Center for Oral Diseases of Zhejiang Province, Key Laboratory of Oral Biomedical Research of Zhejiang Province, Cancer Center of Zhejiang University, Hangzhou, China

**Keywords:** autophagy, natural substances, oral diseases, microbial infection, cancer

## Abstract

Autophagy is a critical factor in eukaryotic evolution. Cells provide nutrition and energy during autophagy by destroying non-essential components, thereby allowing intracellular material conversion and managing temporary survival stress. Autophagy is linked to a variety of oral disorders, including the type and extent of oral malignancies. Furthermore, autophagy is important in lymphocyte formation, innate immunity, and the regulation of acquired immune responses. It is also required for immunological responses in the oral cavity. Knowledge of autophagy has aided in the identification and treatment of common oral disorders, most notably cancers. The involvement of autophagy in the oral immune system may offer a new understanding of the immune mechanism and provide a novel approach to eliminating harmful bacteria in the body. This review focuses on autophagy creation, innate and acquired immunological responses to autophagy, and the status of autophagy in microbial infection research. Recent developments in the regulatory mechanisms of autophagy and therapeutic applications in oral illnesses, particularly oral cancers, are also discussed. Finally, the relationship between various natural substances that may be used as medications and autophagy is investigated.

## Introduction

Autophagy is an evolutionarily conserved cellular process that maintains energy homeostasis through the lysosomal machinery. It is responsible for transporting damaged organelles, misfolded proteins, and other macromolecular material to lysosomes for degradation and reuse (Wei et al., 2018). Autophagy can be divided into six main steps: initiation, vesicle nucleation, membrane elongation, closure, maturation, and degradation (Lapaquette et al., 2015). Several existing studies have revealed that autophagy is triggered to various degrees during the differentiation progression of many cells, such as angiogenesis (Yang et al., 2015), osteogenic differentiation (Damiati and El-Messeiry, 2021), adipogenesis (Deleyto-Seldas and Efeyan, 2021), neurogenesis, and other processes. The autophagy process was discovered in 1963 by the Belgian chemist Christian de Duve. Contemporary correlative research was fueled by studies of yeast in the 1990s through the identification of autophagy-related genes. One scientist, Yoshinori Ohsumi, was awarded the 2016 Nobel Prize in Physiology or Medicine for the discovery of cellular autophagy machinery (Chen et al., 2019). Autophagy is conducive to cellular catabolism and is also a vital defense mechanism against pathogen infection. Autophagosomes capture invading bacteria, deliver them to lysosomes for degradation, and limit the survival and reproduction of pathogens within the cell. However, certain microbes have also evolved various strategies to escape host cell autophagy. For example, some microbes can regulate autophagy, then use the autophagy machinery to form a replicative niche and promote intracellular growth (Brasil et al., 2017). With the progress of cell autophagy in pathogen infection, scholars have found that the interaction between microbes and host cell autophagy is complicated and varied (Lerner et al., 2017). Therefore, a comprehensive and systematic clarification of the molecular mechanisms by which autophagy develops and interacts with microbes will facilitate the understanding of the immune escape mechanisms of infection. Besides, it will provide a theoretical basis and effective therapeutic targets for the treatment of microbial infections. Recent studies have revealed that many oral diseases, such as periodontal disease, oral tumors, and oral mucosal conditions, are closely related to autophagy (Chen et al., 2017). Among them, oral tumors pose the greatest threat to human health. Relevant studies have found that autophagy affects the occurrence and development of oral tumors and regulates the progression of oral tumors through different autophagy signaling pathways. Also, multiple autophagy-related genes have dual regulatory effects on tumors (Chen et al., 2017). The differential expression of autophagy-related genes directly affects the progression of oral tumorigenesis and development (White et al., 2011), while advances in tumor therapeutics targeting the autophagy pathway also provide new ideas for treating oral cancer. Finally, several recent studies have also shown that many natural substances that are effective in disease treatment participate in autophagy regulation to varying degrees, which may exert therapeutic effects by regulating autophagy (Wang and Feng, 2015). After accounting for the various factors mentioned above, this article first reviews the links between oral diseases (especially tumors) and autophagy, then explores some associations between autophagy and microorganisms. Finally, it summarizes some of the possible therapeutic mechanisms of active ingredients from natural substances on diseases and their targets or pathways regulating cell autophagy and explores new targets with the drug candidates.

## Cell autophagy

### Autophagy classification

**FIGURE 1:**
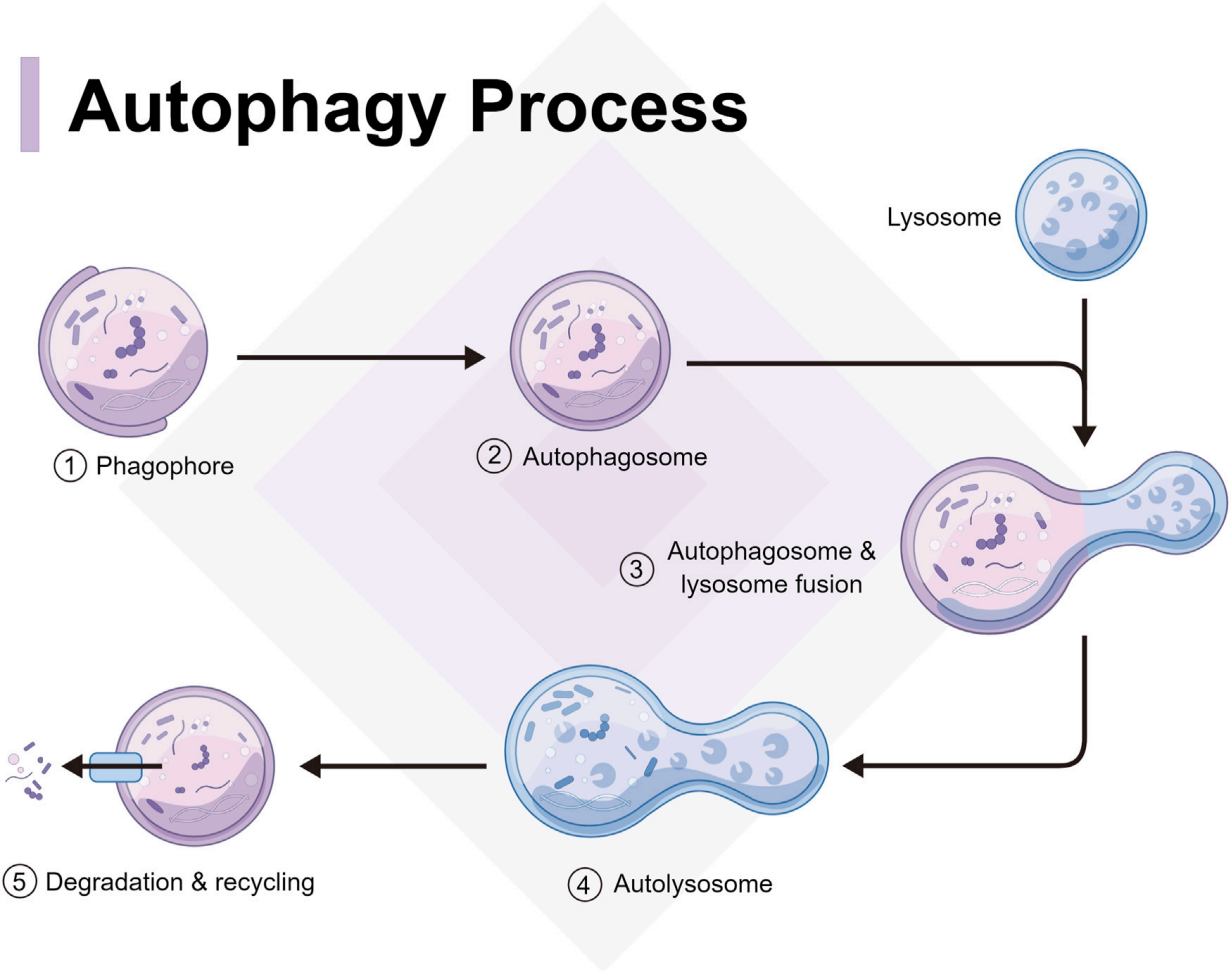
Process and mechanism of autophagy, including autophagy signal induction, precursor formation, phagocytic vesicle extension, autophagosome and lysosome fusion, and autophagolysosome degradation.

Autophagy can be categorized into two forms: classical and non-classical autophagy. Classical autophagy can be further divided into molecular chaperone-mediated autophagy, micro-autophagy, and macroautophagy, according to the substrate features, transport types, and regulatory mechanisms (Theofani and Xanthou, 2021). However, autophagy generally refers to macro-autophagy and in genetics, it is defined as an autophagosome characterized by a double membrane morphological structure in the cytoplasm that fuses with lysosomes (Acevo-Rodríguez et al., 2020). From yeast to mammals, autophagy has always been highly conserved, and the relevant process can be divided into several steps as follows (Figure 1): autophagy signal induction, precursor formation, phagocytic vesicle extension, autophagosome and lysosome fusion, and autophagolysosome degradation (Li et al., 2016a). Non-classical autophagy refers to LC3-related phagocytosis, which mainly targets intracellular macromolecules. Cells isolate pathogens after phagocytosis in an LC3-positive single membrane compartment called LAPosome. LAPosome then transports the pathogens to the lysosomes for degradation and clearance. The autophagy level is closely related to the occurrence and development of infectious diseases (Khandia et al., 2019), while macro-autophagy and LAP play an important role in resistance to pathogen infection.

### Autophagy occurrence and regulation

Autophagosome induction is strictly regulated by a series of signaling pathways (De Palma et al., 2014), including subcellular localization and the post-translational modification of the ATG protein. As an initiation complex of the autophagy signal, the Unc-51-like kinase 1 (ULK1) complex is mostly regulated by two upstream factors, mammalian target of rapamycin (mTOR) and AMP-activated protein kinase (AMPK). Under adequate intracellular nutrition, active mTOR inhibits autophagy through the phosphorylation of ULK1 Ser 757 (Li et al., 2019). When intracellular nutrition deficiency or pathogen infection occurs, mTOR becomes inactive and AMPK initiates autophagosome formation by phosphorylating ULK1 Ser 317 and Ser 777. Meanwhile, the ULK complex regulates phosphorylation and recruitment of Type III phosphatidylinositol 3-kinase complex, thereby promoting the production of phosphatidylinositol 3-phosphate (Wang et al., 2019a). At this point, phagocytic vacuoles begin to form at endoplasmic reticulum-mitochondrion contact sites to complete the synthesis of the phagosome precursor (Liu et al., 2017). Two independent ubiquitin-like connection systems control the production of the ATG12-ATG5-ATG16L1 complex and LC3-PE, which is critical for the elongation and closure of phagocytic vacuoles. The ATG16L complex promotes the formation of LC3-PE and anchors it to the autophagosome membrane. However, ATG4 may partially uncouple the LC3-PE complex and promote the recycling of LC3 to form new autophagosomes (Huang et al., 2021a). Finally, with the participation of multiple proteins such as Rab7, syntaxin 17, vesicle-associated membrane protein 8, and lysosome-associated membrane protein, autophagosomes fuse with lysosomes to form autophagolysosomes. After degradation with peptidase, lipase, and hydrolase, the autophagolysosome contents are recycled into the cytoplasm (Foroozandeh and Aziz, 2018).

## Autophagy and human innate immunity

Autophagy is an essential element of natural immune response, but the signaling pathway for its activation remains unclear.

### Monocytes/macrophages

Autophagy requires the differentiation and activation of monocytes/macrophages (Maiuri et al., 2013). Defects in autophagy may also compromise nitric oxide production, phagocytosis, and the bactericidal ability of macrophages. In macrophages without autophagy, mitochondria and reactive oxygen species (ROS) aggregate, leading to an increase in pro-inflammatory cytokine levels (Barbosa et al., 2018).

### Dendritic cells

**FIGURE 2:**
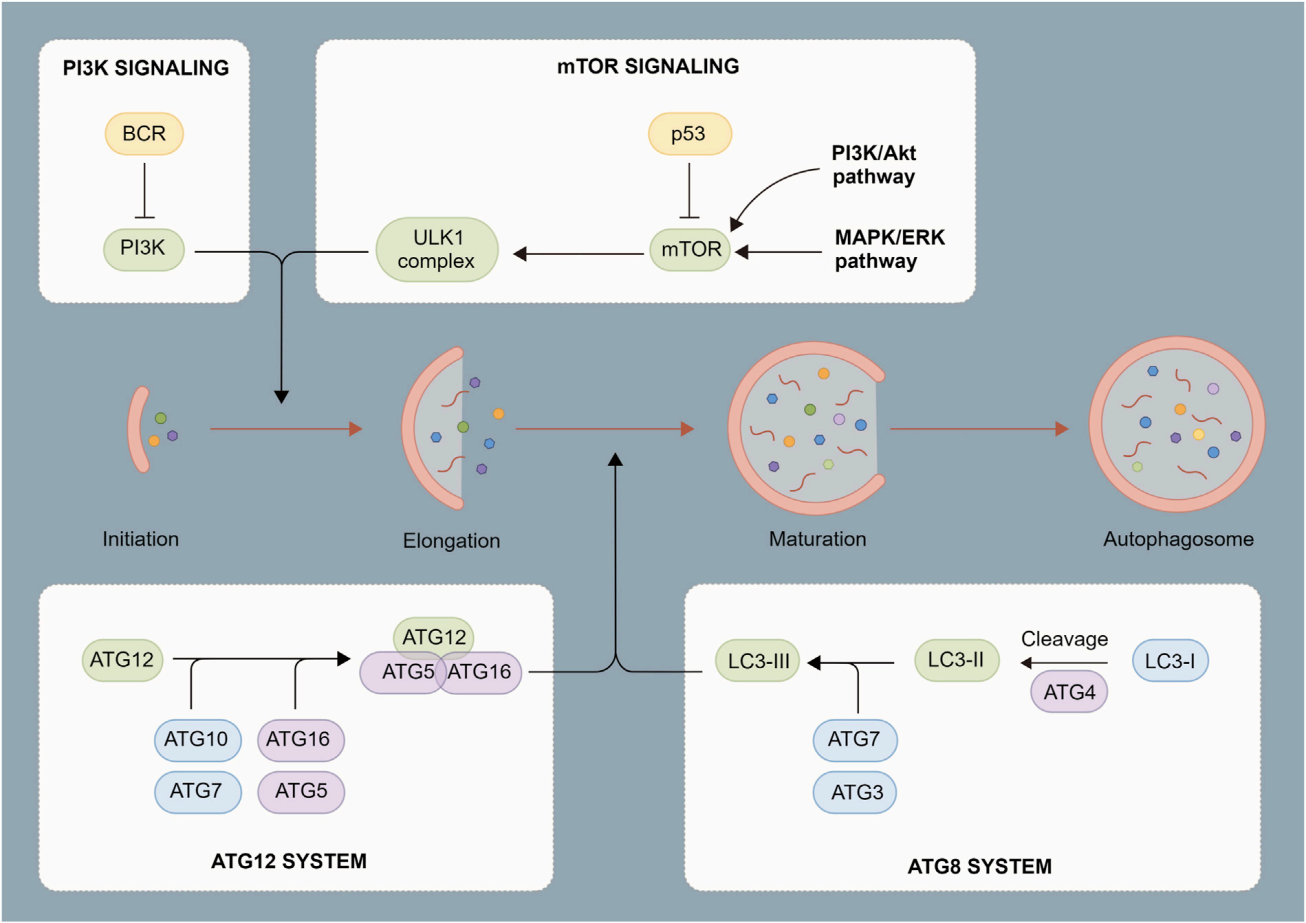
Process of autophagosome formation. Autophagosomes are composed of a double membrane enclosing a small portion of the cytoplasm. The cytoplasm contains digested material consisting of various components such as mitochondria, endoplasmic reticulum fragments, etc. The contents are degraded through fusion with lysosomes.

After infection, dendritic cells (DCs) induce autophagy through activated nod-like receptors and toll-like receptors. Autophagy provides lysosomes with cytoplasm and phagocytic extracellular antigens for further TLR activation, cytokine secretion, and antigen presentation (Allemailem et al., 2021). Mitochondria and reactive oxygen become uncontrolled due to a lack of autophagy, resulting in the aging of DCs (Figure 2).

### Neutrophils

Autophagy is induced by anti-neutrophil cytoplasmic Abs and promotes neutrophil extracellular trap formation (Bravo-Barrera et al., 2017). NETs are antimicrobial extracellular chromatin structures released by neutrophils that have undergone NETosis cell death. Most relevant studies assert that neutrophils in both humans and mice need autophagy to achieve optimal activation (Zhang et al., 2017). First, autophagy is induced when human neutrophils are activated. Also, autophagy defects lead to a series of functional deficiencies, including lower neutrophil degranulation in mice, reduced destruction of intracellular bacteria by human neutrophils, and compromised NET formation (Wang et al., 2019b). Since the function of aged neutrophils is reduced and autophagy is required for the optimal activation of neutrophils, it is necessary to consider whether the autophagy of aged neutrophils is compromised or whether autophagy improves the function of aged neutrophils (Jiang et al., 2019).

## Autophagy and microbial infection

**FIGURE 3:**
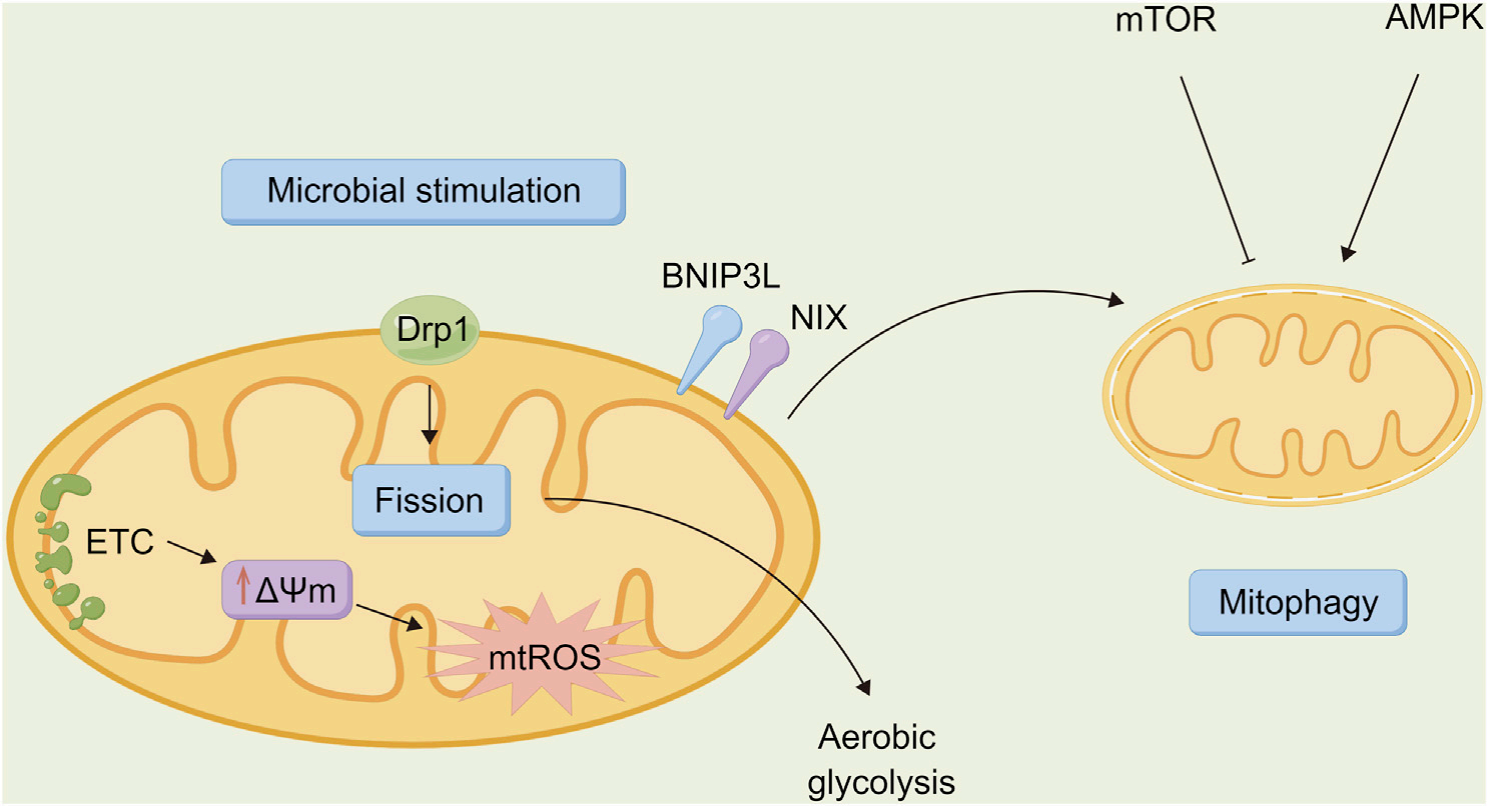
Relationship between autophagy and microbiota. Autophagy is a “double-edged sword” that is involved in host defense against pathogen infection, allowing invading bacteria to be transported to lysosomes for degradation via lipid raft mediated endocytosis. However, it can also be a unique strategy for certain pathogenic bacteria to evade immune surveillance, thereby promoting the survival, proliferation, and extent of infection in the incoming bacteria.

Autophagy acts on immune cells directly to regulate their function so that the body can resist the invasion of pathogens. However, as a “double-edged sword” (Figure 3), autophagy may be utilized by other pathogenic microorganisms to protect the body, thus promoting the infection of host cells and causing damage (Ke, 2018). Several types of pathogenic microorganisms have an intricate relationship with autophagy. Studying the relationship between autophagy and microbial infection may also provide a new direction for further exploring the infection mechanism of pathogenic microorganisms as well as novel methods for fighting infection.

### Autophagy for clearance of microbial infection

Resisting microbial invasion is one of the mechanisms for autophagy. Invading microorganisms are captured directly to form autophagosomes, which fuse with lysosomes (Huang et al., 2021b). Thus, microorganisms are degraded and eliminated successively. Studies have shown that a variety of mechanisms are involved in the regulation of this process, including pattern recognition receptors (PRRs), which are an important way to mediate the capture of invading microorganisms (Steimle and Frick, 2016). PRRs recognize the molecular signatures of microorganisms and then recruit intracellular autophagy devices to capture the invading microorganisms (Shibutani et al., 2015). Another path is based on invading microorganisms consuming nutrients in cells. In other words, autophagy activity may be promoted due to a lack of nutrients in cells, which is conducive to the elimination of invading microorganisms. The third pathway in regulating the activity of autophagy is the interaction of Beclin-1 with TNF receptor-associated factor 6 in the TLR signaling pathway (Wen et al., 2020). To initiate autophagy, the cells first detect whether microorganisms have entered the cytoplasm, then the corresponding locations with PRRs play an important role in microorganism identification (Wang et al., 2020). TLR and NOD-like receptors also sense microbial invasion and initiate autophagy in the early stages of infection. They induce autophagy when the cells engulf the bacteria or at different stages of replication after the cells are infected by viruses, thus facilitating the elimination of viruses. Autophagy is also involved in the resistance to toxoplasma gondii infection (Subauste, 2021).

### Autophagy promoting microbial infection

Some bacteria may develop new mechanisms to evade or exploit autophagy through evolution. After invading host macrophages, *Mycobacterium tuberculosis* blocks the autophagy process by preventing autophagolysosomes from acidification and maturation. Therefore, it escapes the immune response of the body and poses a hidden danger for the incubation of *Mycobacterium tuberculosis* in organisms, thereby causing tuberculosis relapses (Kaleagasioglu et al., 2020). Shigella evades the autophagy of host cells through secretion of the IcsB protein by the Type III secretion system (Sasakawa, 2010). Salmonella and legionella interfere with the maturation of autophagosomes in host cells through the secretion system and survive by inducing the formation of autophagosome-like structures or causing the programmed death of macrophages. *Helicobacter pylori* stimulates macrophages to form autophagosomes and multiplies in double-membrane autophagosomes (Wang et al., 2021a). Autophagy may also be necessary for the growth of some parasites, such as *Trypanosoma cruzi*. By degrading organelles and some senescent proteins, autophagy provides nutrition for the development of *Trypanosoma cruzi*, thus playing an important role in its overall developmental cycle (Mesías et al., 2019). Some viruses may also escape clearance by either inhibiting or utilizing autophagy. Therefore, the resistance of autophagy against viral infection depends on the type and species of the cell. Additionally, autophagy-associated proteins are essential for the replication of the human immunodeficiency virus (HIV), which utilizes the formation of autophagosomes to promote its own replication (Ramesh et al., 2019).

## Autophagy and oral diseases

### Fluorine spot tooth

Fluorine spot tooth is also known as dental fluorosis or mottled enamel. It refers to incomplete enamel mineralization caused by damage to ameloblasts in the enamel mineralization stage due to excessive fluoride intake in the human tooth during its development and mineralization (Wei et al., 2019). It is widely accepted that endoplasmic reticulum stress (ERS) occurs when ameloblasts are stimulated by excessive fluoride, leading to protein expression errors and apoptosis, which are associated with the formation of fluorine spot tooth (Wei et al., 2019). When the mouse ameloblast-derived LS8 cell line is treated with fluoride at different concentrations, the expression levels of endoplasmic reticulum chaperone molecules GPR78 and XBP-1 rise with increasing fluoride concentrations, indicating that fluorosis induces ERS in ameloblasts (Yang et al., 2013). In one experiment, different concentrations of fluoride (0, 1, and 5 mmol/L) were used for 24 h of treatment of the first molars in Balb/c mice aged 1 day. As a result, the expression levels of pro-apoptotic proteins, e.g., Bcl-2 associated X protein (Bax), BH3 domain apoptosis protein, cysteine asparaginase-specific protease 8, and caspase-3, were up-regulated in a concentration-dependent manner. At the same time, the disintegration of ameloblasts and odontoblast-papillary area cells was observed. During tooth development, long-term or chronic fluoride intake allows ameloblasts to remain in ERS or even induces apoptosis. Thus, the early enamel matrix protein and late hydrolytic protease cannot be secreted normally, affecting the final enamel formation to a certain extent (Zhao et al., 2021). This may partially contribute to dental fluorosis. Additionally, Some scholars found that when ameloblasts were given excessive fluoride for intervention, the expression levels of LC3 and Beclin-1 increased with fluoride concentration, indicating that the intervention of excessive fluoride caused increased levels of autophagy. Some scholars have speculated that excessive fluoride may simultaneously cause ERS and autophagy in ameloblasts of developing teeth, thereby playing a role in the occurrence and development of dental fluorosis. However, the mechanism of mutual regulation between the two in the occurrence of dental fluorosis is yet to be fully explored (Tian et al., 2020).

### Periodontal disease

**FIGURE 4:**
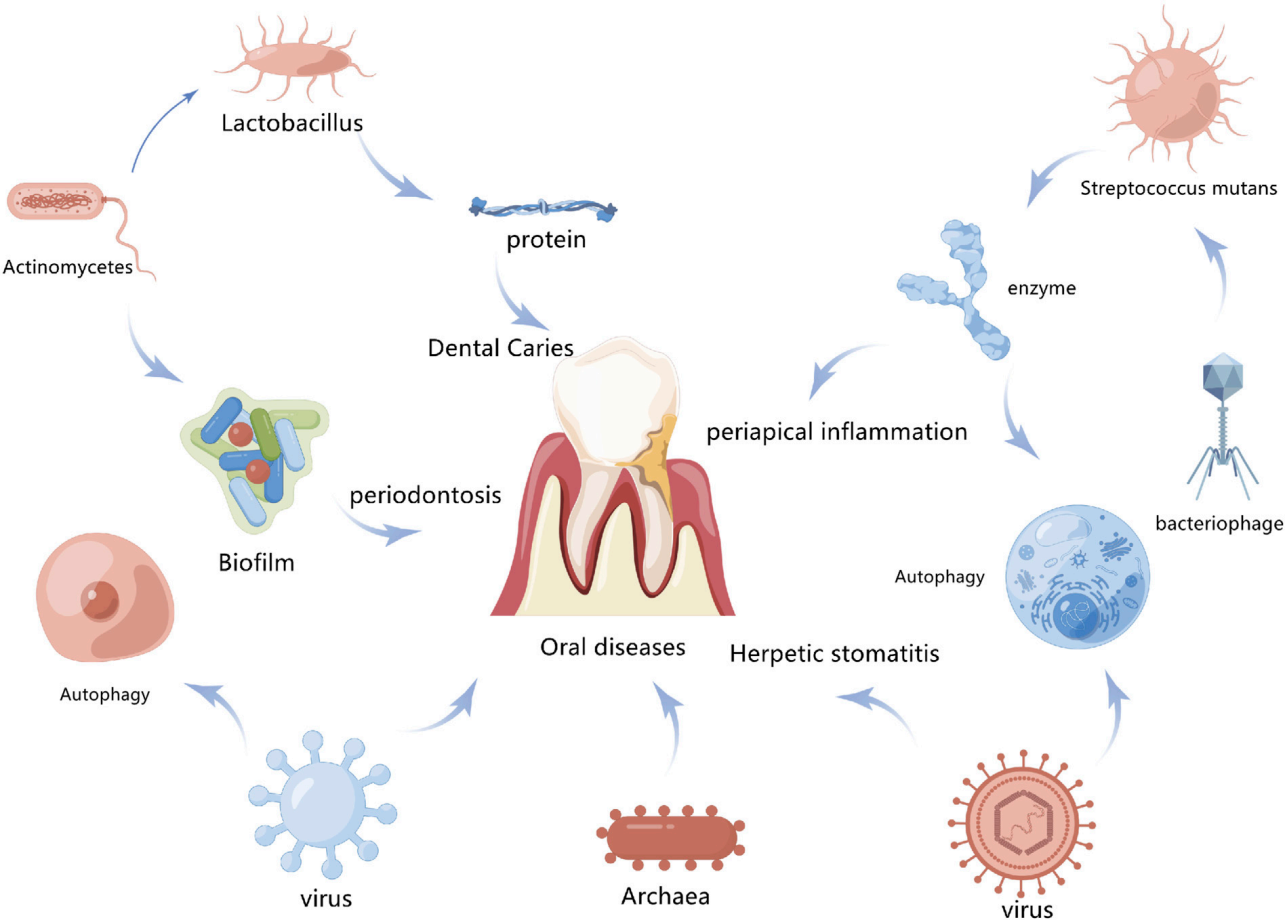
Association between autophagy and oral disease. Autophagy promotes the clearance of bacteria and toxins by infected cells and also contributes to the inhibition of inflammatory responses to maintain intracellular environment homeostasis, which is closely related to the occurrence and development of oral diseases.

Periodontal supporting tissue includes alveolar bone, gum, periodontal membrane, and cementum (Crotti et al., 2015). Periodontitis is a chronic disease that affects the integrity of the tissue that supports teeth. It is also the most common inflammatory disease in periodontal tissue, leading to progressive alveolar bone resorption. Inflammation, as a protective response of the body to injury or microbial infection, is closely related to both ERS and autophagy (Gilroy and Bishop-Bailey, 2019). In periodontitis, periodontal pathogens that carry or release virulence factors like LPS and bacterial DNA interact with toll-like receptors in periodontal cells to activate the autoimmune system, resulting in local inflammatory cell infiltration of tissue and releasing inflammatory factors (da Silva et al., 2019). TLR4 recruits Beclin-1 and competitively binds Beclin-1 with Bcl-2 to reduce the inhibition of Bcl-2. It also stimulates the aggregation of LC3 in the cytoplasm as a feature of autophagy induction. ERS also induces cell death and autophagy in human gingival cells via P38 mitogen-activated protein kinase (P38MAPK) (Wang et al., 2018). In mice, when autophagy genes Atg7 and Atg5 are knocked out, spontaneous aseptic pulmonary inflammation is observed (Arakawa et al., 2017). Inflammation is characterized by the mass recruitment of inflammatory cells associated with an increase in several pro-inflammatory cytokines in broncho-alveoli and serum, which is mainly mediated by interleukin-18 (IL-18). After LPS injection, the levels of pro-inflammatory cytokines in the lungs and serum as well as the mortality of autophagy-deficient mice are higher than in normal mice, indicating that autophagy negatively regulates the inflammatory response (Lei et al., 2019). However, the specific regulatory mechanism of autophagy in these inflammatory diseases is still unclear, and periodontitis is associated with many systemic diseases. Therefore, the relevant mechanism must be further explored (Figure 4).

### Sjogren syndrome

As an autoimmune disease, Sjogren syndrome (SS) targets specific secretory epithelial tissue such as salivary and lacrimal glands, uncommon bronchioles, bile ducts, and distal renal tubules (Rizzo et al., 2019). Regarding the initiation and development of immune attacks, it has been hypothesized that secretory epithelial cells activated after chronic or acute external or internal injury cause the expression of SS-associated antigen A (SSA) and SS-associated antigen B (SSB) on the surface of cell membranes, resulting in an autoimmune response (Vletter et al., 2020). SS is a disease characterized by CD4+T cell infiltration, in which salivary gland epithelial cells present major histocompatibility complex II to CD4+T cells as antigen-presenting cells, thereby inducing the immune response (Vletter et al., 2020). An evaluation of protein levels in the saliva of SS patients revealed that lysosomal protease cathepsin D, a protein with relatively high concentrations, increases under autophagy. This suggests that many salivary epithelial cells in SS patients are in a state of autophagy. Autophagy has recently been identified as an antigen presentation causing polarization of T helper cell type 1 (Th1) and type 17 through the involvement of MHC II molecules in cytokine secretion (Münz, 2012). Clinically, non-specific autophagy-lysosomal inhibitors like chloroquine have been used to treat different autoimmune diseases. Understanding the complex interaction between autophagy and autoimmunity may help to develop more effective or specific therapeutic strategies (Jones et al., 2020).

## Promotion of autophagy in oral tumorigenesis and its mechanism

**FIGURE 5:**
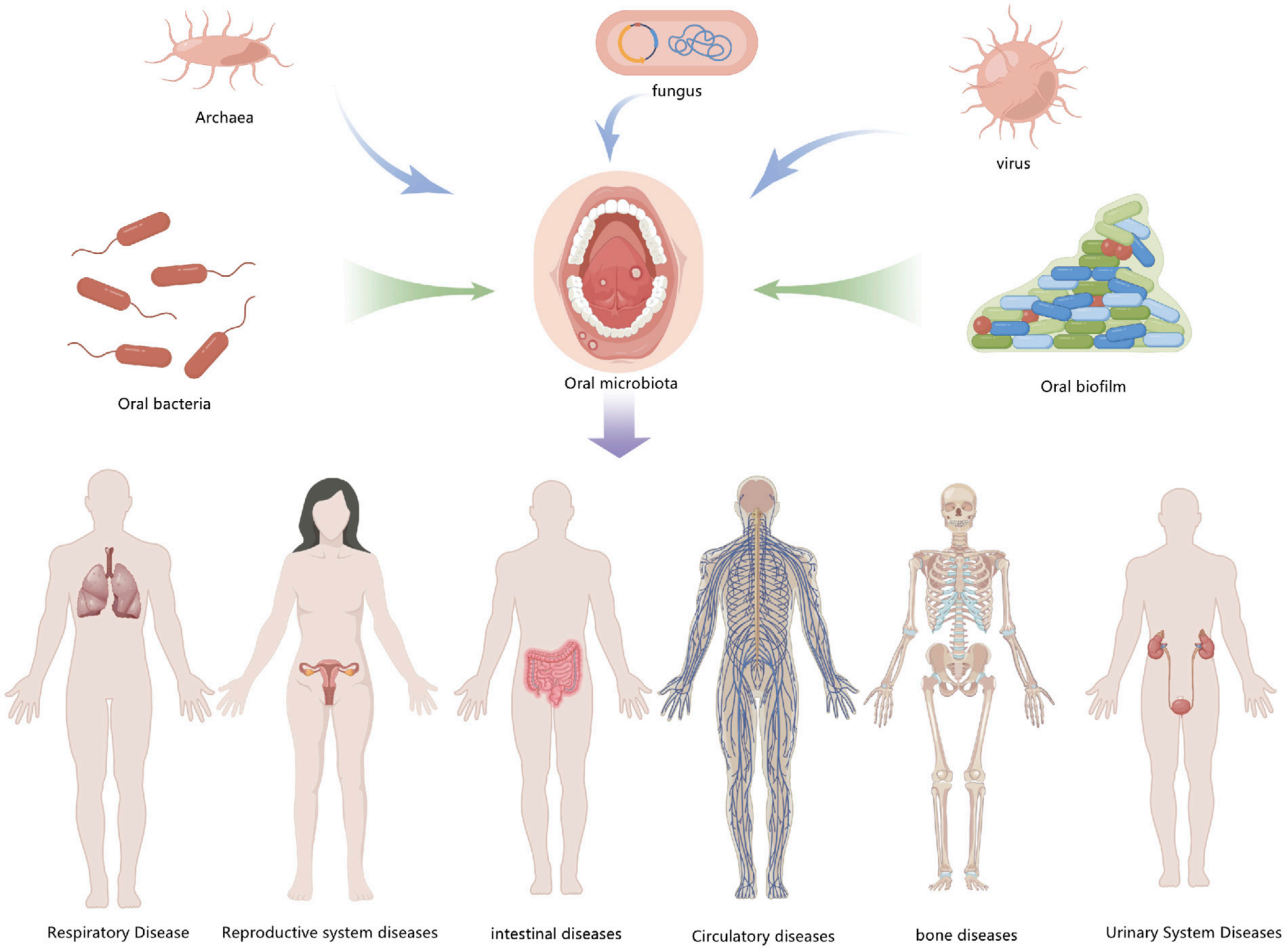
Exploring oral diseases and autophagy may provide references for the study of diseases elsewhere in the body.

Oral health and systemic health are closely related, and modern medicine has demonstrated that periodontitis and other oral diseases are linked to cardiovascular disease, diabetes, cancer, respiratory disease, Alzheimer’s disease, and so on. Exploring the relationship between oral diseases and autophagy is therefore valuable for understanding other systemic diseases in the human body (Figure 5).

### Promotive effect of autophagy on the development of oral carcinoma

The proteins involved in autophagy are known as autophagy-related genes (ATG). Besides, ATG8 (Fernández-Sanz et al., 2019), known as microtubule-associated protein 1 light chain 3 (MAP1LC3 or LC3 for short), is critical for autophagy. In cells of oral squamous cell carcinoma (OSCC), ATG7 participates in the covalent binding of ATG5 and ATG12 in the autophagosome membrane extension stage as an E1-like ubiquitination activase. As a result, the migration of autophagy tumor cells may be inhibited (Nitta et al., 2019). The mTOR protein kinase, a major negative regulator of autophagy (Viana et al., 2018), is involved in several signaling pathways that control cell growth, most of which are downstream of growth factor receptors with tyrosine kinase activity. Structural activation of these receptors, activation mutations of RAS, PI3K, AKT, and inactivation mutations of negative regulators such as PTEN often occur in the progression of cancer. This suggests that the inhibition of autophagy may promote tumor growth (Poillet-Perez and White, 2019). Some studies have emphasized that knocking out the neutrophil gelatinase-associated calcitonin (NGAL) gene induces mTOR activation, thereby inhibiting autophagy and promoting the development of oral tumors (Zhang et al., 2016). In the later stages of tumor evolution, activated autophagy promotes tumor growth by providing metabolically stressed tumor cells with nutrients. Besides, in the early stages of oral cancer, the long non-coding RNA FLJ22447 inhibits the autophagy of tumor-associated fibroblasts (CAFs) and impairs the autophagic degradation of IL33. Also, CAFs release a large amount of IL33 to promote the proliferation of OSCC cells (Saha et al., 2021).

### ROS-dependent NUPR1-mediated autophagy and oral carcinoma

The stress-induced chromatin-related factor NUPR1 is overexpressed in tumor tissue, especially in cases with poor prognosis and high drug resistance (Emma et al., 2016). Also, NUPR1 is essential for the expression of genes associated with metabolic stress response, in particular for those involved in DNA repair, cell cycle regulation, apoptosis, dentate division, and autophagy (Mu et al., 2018). The migration and invasion of oral squamous cell carcinoma cells induced by repeated cadmium exposure may be prevented by inhibiting ROS/NURP1-dependent autophagy (Fan et al., 2019). Additionally, some studies have demonstrated that ROS-dependent NUPR1-mediated autophagy stimulates the proliferation of OSCC cells during repeated cadmium exposure.

### MicroRNA-mediated autophagy and oral carcinoma

MicroRNAs are a group of endogenous RNAs without protein-coding capacity. They affect tumor growth through the action of oncogenes or tumor suppressors (Wozniak et al., 2016; Song et al., 2018; Gao et al., 2020) and inhibit autophagy by targeting autophagy-related genes, thereby promoting the occurrence of oral carcinoma. Pang et al. (2019) observed autophagy that was induced by the hypoxia of CAL-27 cells in human tongue squamous cell carcinoma (TSCC) with a transmission electron microscope. Results indicated that miR-17-5p overexpression limited the Beclin-1 mRNA and protein expression in CAL-27 cells, thereby inhibiting autophagy and promoting tumorigenesis.

### Long Non-coding RNA and oral carcinoma

LncRNAs are divided into carcinogenic lncRNAs and anticancer lncRNAs, according to their effects on tumors (Li et al., 2016b; Dong et al., 2018). In autophagy regulation, lncRNAs form a network through interactions and connections between multiple pathways, thereby jointly regulating the growth and death of tumor cells. Yang et al. (Li et al., 2016b) found that overexpression of the lncRNA CASC9 activated the AKT/mTOR signaling pathway. As a result, autophagy and autophagy-mediated apoptosis are inhibited and the occurrence and development of oral carcinoma are promoted. This suggests that CASC9 is a potential biomarker for oral carcinoma diagnosis and also a potential site for targeted therapy.

## Mechanism of the inhibitory effect of autophagy on oral tumorigenesis and development

Autophagy has a dual function in oral carcinoma (Yang et al., 2019). The inhibition of autophagy may promote the proliferation, invasion, and migration of oral cancer cells, thereby advancing the occurrence and development of oral cancer. Meanwhile, the autophagy of oral cancer cells can be enhanced by targeted drugs through chemotherapy or radiotherapy, preventing the manifestation or progression of oral cancer (Kardideh et al., 2019). Up-regulation or over-expression of the AKT/mTOR pathway leads to tumor growth and poor prognosis by affecting autophagy. Besides, the MAPK signaling pathway involved in the proliferation, differentiation, apoptosis, angiogenesis, invasion, and metastasis of tumor cells plays an important role in oral cancer (González et al., 2021). Therefore, by inhibiting activation of the MAPK signaling pathway, the occurrence of oral cancer may be suppressed. Studies have shown that fern diene induces autophagy in human OSCC cells by regulating the Akt and MAPK pathways. Besides, Bi-d1870, a specific inhibitor of downstream targets in the MAPK signaling pathway (Penisson et al., 2019), inhibits oral carcinoma by regulating the expression of cell cycle regulators like P21 and inducing cell cycle arrest in the G2/M phase. Studies have established that cancer suppressor genes controlling the autophagy pathway include PTEN, Beclin-1, and DAPK. The activated PTEN may promote downregulation of the PI3K/AKT pathway, affecting tumor growth by stimulating autophagy (Youn et al., 2021). As one of the most important autophagy regulators, Beclin-1 acts as a tumor suppressor mammalian cell. DAPK, a phosphorylated Beclin-1 kinase, is also known as a death-related protein kinase that disrupts the Beclin-1/Bcl-2 complex (Casterton et al., 2020; Kaushal et al., 2020). According to some studies, the DAPK gene provokes cancer development by inhibiting autophagy as an autophagy inducer inhibition in different types of human cancers.

## Autophagy in oral cancer therapy

Autophagy is a dynamically balanced catabolic degradation process, in which cellular proteins and organelles are engulfed by autophagosomes, digested by lysosomes, and then recycled to maintain cellular metabolism (Watada and Fujitani, 2015). A lower OSCC cell autophagy ability reflects a higher degree of malignancy and chemotherapy resistance. Sensitivity to cell death may be improved by combining chemotherapy with autophagy inhibitors, suggesting that OSCC responds better to autophagy-based therapy. Additionally, OSCC cell autophagic death may occur during radiation. At the same time, some drugs induce the autophagy of OSCC cells, thereby treating OSCC. Several studies related to autophagy have provided a sound experimental basis for OSCC treatment, so utilizing autophagy to treat OSCC has become the focus of much current research (Adnan et al., 2019).

### Using CerS6 to enhance sensitivity to cisplatin chemotherapy in OSCC treatment

To determine whether autophagy regulates drug resistance in oral cancer chemotherapy, some scholars (Li et al., 2018; Zhang et al., 2019; Krasikova et al., 2021) explored the effects of CerS6 on autophagy and mitochondrial fusion of chemotherapy-resistant cells using models of cisplatin-resistant OSCC cells and xenograft in nude mice. These studies revealed that high expression of CerS6 enhanced mitochondrial cleavage and apoptosis in cisplatin-resistant OSCC cells, and attenuated cisplatin-induced autophagy. Also, CerS6 may increase the sensitivity to cisplatin by altering the expression of calpain. The xenotransplantation of cisplatin-resistant OSCC cells into a nude mouse model demonstrated that CerS6 increased the sensitivity to cisplatin-resistant chemotherapy, and thus, the tumor volume was reduced.

### Regulating autophagy-related genes to inhibit the development of oral carcinoma

As a histone methyltransferase, G9a is closely related to DNA methylation. In recent years, studies have shown that G9a affects oral carcinoma by regulating autophagy response. Also, G9a may combine transcription factors to participate in the occurrence of various diseases (Caffrey et al., 2016). Maeda et al. (Ahmad et al., 2016) discovered that G9a promoted the autophagy of OSCC cells and reduced the development of OSCC by inhibiting H3K9 methyltransferase. Sambandam et al. (Maeda et al., 2017) found that OSCC cells highly expressed with RANKL significantly increased the expression of autophagy-related genes LC3, ATG5, BECN1, and PI3KC3. A further study demonstrated that RANKL induced the formation of autophagosomes, providing evidence for the targeted therapy of oral carcinoma. Wang et al. (Sambandam et al., 2016) enhanced the autophagy of OSCC cells by treating OSCC cells with erianin. As a result, erianin reduced the vitality of OSCC cells, demonstrating its potential as an anticancer drug. Utaipan et al. (Wang et al., 2016) demonstrated that hordenine induced endoplasmic reticulum stress and triggered the p38MAPK-mediated apoptosis of drug-resistant cells and associated autophagic cell death in OSCC. With potential cytotoxic effects, isohordenine may provide potential antitumor effects against multi-drug-resistant oral carcinomas.

### Regulating autophagy-related non-coding RNA to inhibit oral carcinoma

The development of non-coding miRNAs that bind to the 3′UTRs of target genes for translation inhibition or mRNA degradation suggests that it is possible to treat oral carcinoma using miRNAs with autophagy as the targeted pathway (Utaipan et al., 2017). Studies have shown that the low expression of miRNA-137 in OSCC is related to the degree of tumor differentiation, and miRNA-137 is expected to be a potential marker for the early diagnosis of OSCC (Shree Harini and Ezhilarasan, 2022). The study of Zhu et al. (Sun and Li, 2018) showed that miRNA-29b suppressed osteosarcoma cells by targeting CDK6 and inhibiting cell proliferation, migration, and invasion.

## Autophagy induced by natural drugs

**FIGURE 6:**
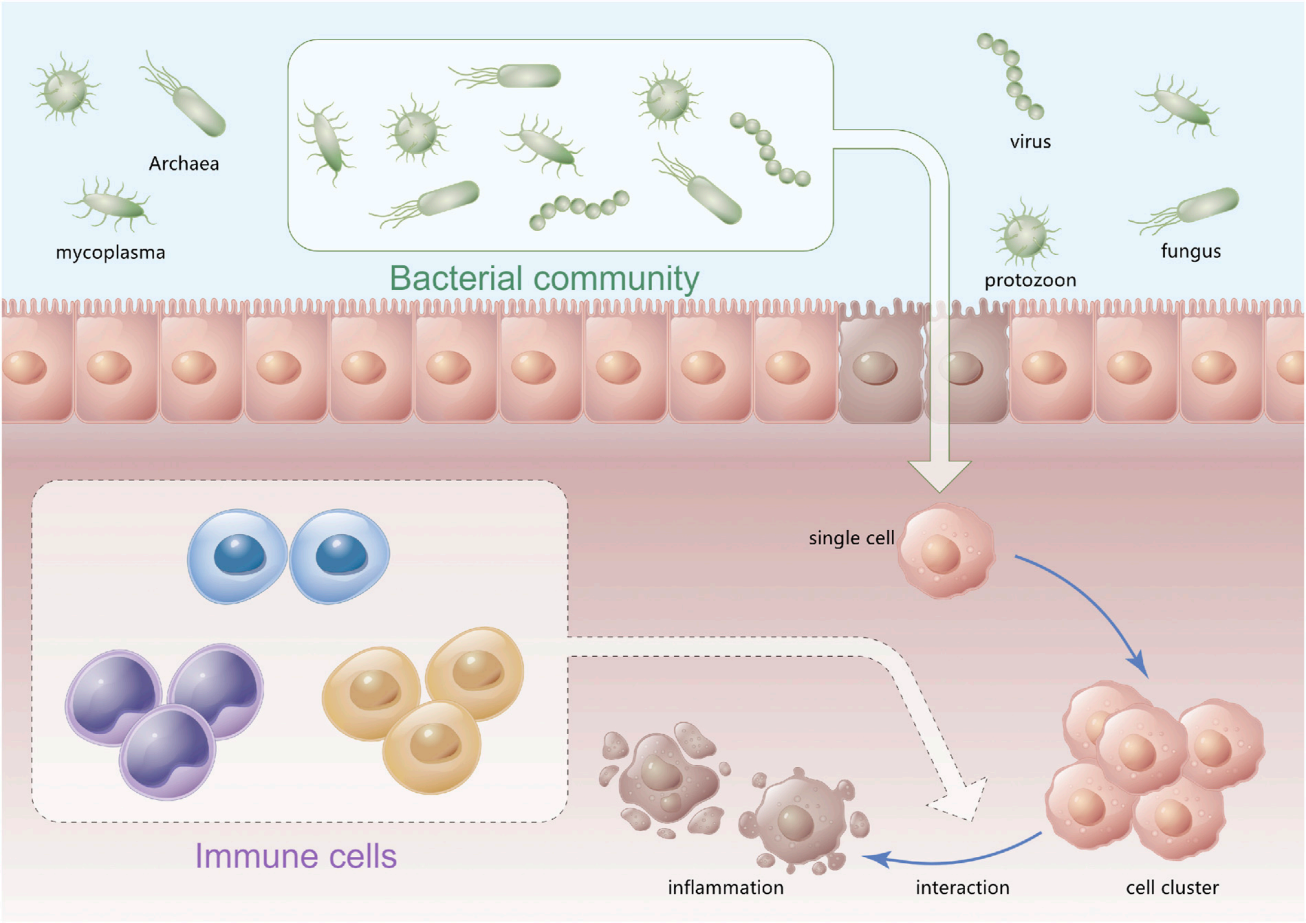
Relationship between immune cells and microorganisms.

The therapeutic properties of chemicals isolated from natural products have been applied in the treatment of various diseases (Song et al., 2017; Zhu et al., 2021a; Wang et al., 2021b; Zhu et al., 2021b; Wen et al., 2021; Zhang et al., 2021; Bai et al., 2022). In recent years, the activation of autophagy by natural substances has gained increasing attention (Table 1; Figure 6). As an extract of *Reynoutria japonica*, *Arachis hypogaea*, *Vitis vinifera*, and *Fructus mori*, resveratrol is characterized by various biological activities and pharmacological effects, especially its anti-proliferation effect on tumors (Zhu et al., 2016). Research confirmed that low doses of resveratrol reduced colon tumor progression more effectively than high doses in subjects exposed to a high-fat diet (De Amicis et al., 2019). Besides, resveratrol suppresses the growth of cancer stem-like cells by inhibiting fatty acid synthase (Mouhid et al., 2017). An *in vitro* cell culture assay showed that increased uptake of resveratrol-modified liposomes by hepatocellular carcinoma (HepG2) cells led to better tumor cell killing ability compared with free resveratrol (Palomeras et al., 2018). Resveratrol induces autophagy by directly inhibiting mTOR through ATP competition (Kashapov et al., 2021), which may be related to its excellent anti-tumor effect. Curcumin is the primary curcuminoid in the rhizomes of *Curcuma longa* and is a constituent of the herbs and spices used in traditional Asian cooking (Kashapov et al., 2021). In the subcutaneous xenograft model of U87-MG cells, curcumin significantly inhibited tumor growth and induced autophagy (McBean et al., 2017; Wang et al., 2019c). Some scholars observed that curcumin induced caspase-3-dependent apoptosis and autophagy in Mtb-infected macrophages (Ravindran et al., 2009). While curcumin induces autophagic cell death in HCT116 human colon cancer cells by inducing ROS production (Gupta et al., 2017), it has also exhibited some defects in clinical applications. Higher concentrations of curcumin were observed in normal tissue than in malignant colorectal tissue in patients receiving 3.6 g/day curcumin, with trace levels of curcumin in peripheral blood circulation (Gupta et al., 2020). Bufalin is a key compound found in chansu and huachansu, as well as the toxins of other toad species such as *Bufo marinus* (Tomeh et al., 2019). It is an effective cardiotonic agent, anesthetic agent, blood pressure regulator, and anti-tumor drug, and it induces the autophagy of various human hepatoma cells (Qi et al., 2018). For instance, bufalin induces cell autophagy and inhibits the proliferation of liver cancer cells by influencing the expression of autophagy-related proteins including LC3-I, LC3-II, P62, and Beclin-1 in liver cancer cells. Furthermore, the autophagic state of liver cancer cells affects the inhibitory effect of bufalin in the proliferation of liver cancer cells (Sheng et al., 2021). Additionally, after constraining the autophagy of HCC-LM3 cells, bufalin significantly enhances inhibition of the adhesion, migration, and invasion of HCC-LM3 cells. Also, synergistic inhibition is strongest when different autophagy inhibitors are combined with 3 MA and CQ. After inhibiting autophagy, bufalin significantly inhibits the protein expression of P-AKT, Cyclin D1, MMP- 2, MMP-9, and VEGF in HCC-LM3 cells. Besides, the protein expression of PTEN and E-cadherin in HCC-LM3 cells increases significantly (Qi et al., 2018). Furthermore, bufalin simultaneously activates the autophagy and apoptosis of hepatoma and gastric cancer cells, and blocking bufalin-induced autophagy may aggravate the death of apoptotic cells (Sheng et al., 2018). Ginseng total saponin is a key ginseng extract with many effective components and strong anti-tumor activity, and it is the prime component responsible for the pharmacological effects of ginseng (Lv et al., 2021a). A previous study illustrated that Ginsenoside Rh4 triggered apoptosis and autophagy by activating the ROS/JNK/p53 pathway in colorectal cancer cells, providing evidence that Rh4 shows great potential as an anti-cancer agent (Liu et al., 2019). In another study, Ginsenosides (TGNs) were found to enhance autophagy by promoting acidic vacuole organelle formation, recruitment of enhanced green fluorescent protein-microtubule-associated protein light chain 3, and expression of autophagy-related factors in cervical cancer cell lines. These results prove that TGN has become a promising drug candidate for cancer therapy (Wu et al., 2018). Ursolic acid, a bioactive ingredient isolated from *Radix Actinidiae chinensis*, has a strong antitumor effect on osteosarcoma cells. It inhibits tumor cell proliferation and promotes the apoptosis of a variety of osteosarcoma cells. In a mouse osteosarcoma xenograft model, low-dose cisplatin combined with ursolic acid significantly reduced tumor growth, which is a process that may be associated with autophagy (Bian et al., 2021).

**TABLE 1:** Summary of drugs and mechanisms by which active ingredients and compounds of TCMs activate autophagy.

Drug	Cellular or animal models	dosage	Pathways that activate autophagy
Hyperthyroidism	M22 induces nthy - ori-3–1 in human thyroid follicular epithelial cells	Medicated serum was administered for 24 h *in vitro*	Inhibits the mTOR/P70S6K pathway, increases lc3ii, ATG5 expression, promotes autophagosome formation, and inhibits their degradation
Polysaccharide from Cordyceps pupae	High glucose induced podocytes in mice	25 mg/L *in vitro* for 48 h	Inhibition of JAK/STAT pathway and increased lc3ii, Beclin1, ATG5, atg12 expression
Panax ginseng notoginseng Chuanxiong Hort	High glucose and high fat induced human aortic endothelial senescent cell HAEC	200 mg/L *in vitro* for 24 h	Increased LC3, suppressed p62 expression and promoted autophagosome formation
Guben invigorating blood circulation complexing agents	High glucose stimulates podocyte MPC in podocytes	Medicated serum (compound gavage for 7 days) was administered for 48 h *in vitro*	Increased Atg7, atg12-atg5 expression and promoted autophagosome formation
Tongxin collaterals	Rat model of myocardial ischemia-reperfusion injury	0.5 g/kg body weight for 7 days	Activates the PINK1/parkin mitophagy pathway, increases lc3ii/lc3i, suppresses p62 expression and increases autophagosome number
puerarin	Cadmium induced AML-12 in mouse hepatocytes	200 μ Mol/L *in vitro* for 12 h	Inhibition of ROS, reduced lc3ii, p62 expression and reduced autophagosome accumulation
Formononetin	Human hepatoma HepG2 cells	20 μ Mol/L *in vitro* for 24 h	The AMPK/TFEB pathway is activated to increase lc3ii and p62 expression and promote the fusion of autophagosomes with lysosomes
Tongluo Xingnao effervescent tablets	β Human neuroblastoma cells SH-SY5Y induced by 25–35	15 μ Mol/L *in vitro* for 24 h	Inhibition of mTOR/TFEB pathway, suppression of lc3ii/lc3i, p62 expression, and restoration of lysosomal function
Paeoniflorin and saikosaponin A	Corticosterone induced PC12 in human astrocytes	400 μ Mol/L paeoniflorin and 10 μ Mol/L saikosaponin a for 24 h *in vitro*	Inhibition of mTOR pathway, reduced lc3ii/lc3i, p62 expression and decreased the number of autophagosomes
Yi Huang Tang	Human vaginal epithelial cells infected with herpes simplex virus type 2 VK2/E6E7	15 μ Mol/L *in vitro* for 24 h at 5	Inhibition of PI3K/Akt/mTOR pathway, increased lc3ii, ATG5 expression and restored lysosomal function
berberine	High glucose induced H9c2 in rat cardiomyocytes	100 μ Mol/L *in vitro* for 30 min	Activation of AMPK/mTOR pathway increased lc3ii, Beclin1, ATG5 expression
Tanshinone IIA	Doxorubicin induced H9c2 cells	20 μ Mol/L *in vitro* for 24 h	Activation of Beclin1/lamp1 pathway reduces lc3ii and p62 expression and promotes autophagosome formation and lysosomal degradation

## Inhibition of autophagy by natural substances

Certain natural substances may have an inhibitory effect on autophagy. In human glioblastoma LN229 and U251 cells, peiminine blocks the cell cycle by up-regulating the expression of LC3 and p62. It also inhibits autophagy through the AMPK/ULK1 pathway, which decreases the degradation of autophagosomes (Tang et al., 2021). ADCX is an extract from *Cimicifugae Rhizoma*. After treatment with metformin, an AMPK activator, the expression of p62 is down-regulated and autophagy is activated, which indicates that the AMPK target is vital for the inhibition of peiminine-induced autophagy (Zhao et al., 2018). Besides, ADCX promotes the apoptosis of HepG2/ADM cells by inhibiting autophagy flux. Additionally, ADCX is decisive in the inhibition of autophagic degradation by activating Akt and inhibiting CSTB (Sun et al., 2017). Astragaloside II is a natural substance that significantly reduces the expression of LC3-II and Beclin-1 in a dose-dependent manner. Astragaloside II (80 μm) further decreases the formation of LC3-II, Beclin-1, and GFP-LC3 puncta dots stimulated with 5-fluorouracil (0.2 mm) in Bel-7402/FU cells (*p* < 0.05). Moreover, Astragaloside II sensitizes cells to 5-fluorouracil-induced cell death via the inhibition of pro-survival autophagy involvement in the MAPK-mTOR pathway (Sanduja et al., 2016). Also, it inhibits autophagy by affecting lysosomal function and restores chemotherapy sensitivity to cisplatin through the PI3K/AKT/mTOR pathway. Furthermore, it reduces the survival rate of tumor cells, blocks them in phase S, and ultimately accelerates tumor cell death. Physakengose G (PG) is a new compound that was first isolated from *Physalis alkekengi var. franchetii*, an anticarcinogenic traditional Chinese medicine. PG showed promising anti-tumor effects in a previous study (Wang et al., 2017a), where it inhibited cell proliferation and induced apoptosis in human osteosarcoma cells. Also, PG treatment blocks EGFR phosphorylation and suppresses epidermal growth factor (EGF)-induced activation of downstream signaling molecules such as AKT and mTOR. PG treatment leads to lysosome dysfunction by altering lysosome acidification and LAMP1 levels, which causes autophagosome accumulation and autophagic flux inhibition. Besides, PG inhibits cell proliferation and EGFR/mTOR signaling in human osteosarcoma cells. Moreover, it induces apoptosis through the mitochondrial pathway and impedes autophagic flux via lysosome dysfunction (Wang et al., 2017a). Furthermore, PG changes lysosome acidification and LAMP1 levels to induce lysosome dysfunction, which causes autophagosome accumulation and autophagy inhibition, thereby inducing apoptosis. Sporoderm, which is the broken spores of *Ganoderma lucidum* water extract (BSGLWE), induces the autophagy activation of osteosarcoma cells and increases the number of autophagosomes blocking autophagy (Lin et al., 2018). It induces the apoptosis of osteosarcoma cells by inhibiting their proliferation and migration. In human colon cancer HT-29 and HCT116 cell lines, PG stimulates lysosome acidification and decreases cathepsin activity. This subsequently blocks the autophagosome-lysosome fusion process, thereby significantly inhibiting autophagy. Moreover, PG causes the accumulation of autophagosomes through the MAPK/ERK pathway and promotes the apoptosis of tumor cells.

## Effect of mixed natural drugs on autophagy

Chinese herbal compounds are composed of more than two herbal medicines and are processed and administered according to regular methods (Wang et al., 2017b). These compounds for the treatment of diseases and syndromes constitute a major component of traditional Chinese medicine prescriptions (Shan et al., 2020). Tongluo Xingnao effervescent tablets restore the function of autophagolysosomes by inhibiting the mTOR/TFEB pathway in human neuroblastoma cells. They also activate autophagy to degrade autophagic vesicles and play a role in resisting Alzheimer’s disease (Wang and Wu, 2020). Additionally, Yi Huang Tang activates autophagy to resist HSV-2 infection by inhibiting the PI3K/AKT/mTOR pathway (Li et al., 2021). However, PI3K inhibitors prevent autophagy activation induced by Yi Huang Tang, indicating that this decoction activates PI3K targets related to autophagy flux. Guben Huayu Tongluo reduces urinary protein excretion and relieves renal pathological damage. Wnt4, p-GSK3β (S9), and β-catenin expression are decreased in the signaling pathway by Guben Huayu Tongluo, but GSK3β levels do not change in diabetic rats. Furthermore, the expression of TGF- and ILK decreases, but the level of E-cadherin increases in diabetic rats after treatment with HTH (Duan et al., 2020). Besides, Guben Huayu Tongluo activates autophagy by promoting the formation of autophagosomes, thereby improving the abnormal autophagy of podocytes under a high-glucose environment and alleviating the damage to podocytes induced by high glucose. In M22-induced human thyroid follicular epithelial cells (Nthy-ori 3-1), Jiakangning capsules containing serum stimulate autophagy and promote apoptosis by activating the AMPK/mTOR pathway (Bai et al., 2017). This indicates that AMPK targets play a vital role in activating autophagy and inhibiting the excessive proliferation of thyroid epithelial cells. In a hypoxia/reoxygenation model related to rat H9C2 cardiomyocytes, Tong Xin Luo activated autophagy flux through the PINK1/Parkin mitochondrial autophagy pathway. It eliminates damaged mitochondria and treats the degradation of misfolded protein through cooperation with the ubiquitin-proteasome system, exerting a protective effect on cardiomyocytes. Table 2 lists the active ingredients of herbal medicines and compounds that inhibit autophagy. Yiqi Huayu Jiedu (YQHYJD) blocks the autophagy of SMCC-7721 by inhibiting the PI3K/mTOR signaling pathway, increasing the number of autophagosomes, and promoting the death of tumor cells, thus playing a role in resisting tumors (Li et al., 2015). Congrong Tusizi Wan inhibits the activation of autophagy by preventing the expression of LC3 and Beclin-1 through the PI3K/Akt/mTOR pathway and reducing the number of autophagosomes and autophagolysosomes in rat ovarian granulosa cells. (Wu et al., 2017). In a lipopolysaccharide-induced macrophage activation model, the Buyang Huanwu decoction inhibited the autophagy of macrophages and the expression of LC3 and p62 by selectively blocking the PI3K/Akt/mTOR pathway (Lv et al., 2021b). This reduces macrophage autophagy and inflammatory reactions and stabilizes vulnerable atherosclerotic plaques. Also, in mouse glomerular podocytes induced by azithromycin, Modified Huangqi Chifeng Decoction (MHCD) inhibited the initiation of autophagy and the combination of autophagosomes and lysosomes through the ROS-autophagy pathway, thus blocking autophagy and protecting glomerular podocytes (Yu et al., 2018; Zheng et al., 2021).

**TABLE 2:** Summary of drugs and mechanisms by which active ingredients and compound compounds of traditional Chinese medicine inhibit autophagy.

Drug	Cellular or animal models	Dosage	Inhibition of autophagy signaling pathway
Astragaloside II	Cisplatin induced sgc-790 human gastric cancer cells, HepG2 human hepatocellular carcinoma cells, smc-7721 cells	50 μ Mol/L *in vitro* for 30 min	Activates the PI3K/AKT/mTOR pathway, increases the expression of LC3II and p62, and inhibits lysosomal function
Physakengose G	Human osteosarcoma u-2os cells	15 μ Mol/L *in vitro* for 12 h	Inhibition of EGFR/mTOR pathway, increased lc3ii, p62 expression and inhibited lysosomal degradation
Huangqi Chifeng Decoction	Doxorubicin induced podocytes in mouse glomeruli	Medicated serum (compound gavage for 7 days) was administered for 24 h *in vitro*	ROS pathway inhibited lc3ii/lc3i, Beclin1 expression, increased p62 expression and inhibited lysosomal function
buyang huanwu decoction	Lipopolysaccharide induced RAW264.7 macrophages	Medicated serum (compound gavage for 7 days) was administered for 24 h *in vitro*	Activated PI3K/Akt/mTOR pathway, inhibited lc3ii/lc3i, p62 expression and reduced autophagosome number
Yiqi invigorating and detoxifying formula	Human hepatoma cells smcc-7721	5 g/L *in vitro* for 24 h	Inhibition of PI3K/mTOR pathway increased lc3ii, Beclin1, p62 expression, leading to the impairment of autophagosome degradation
Beomyosin B	Human glioblastoma cells ln229 and U251	200 μ Mol/L *in vitro* for 7 days	Inhibition of AMPK/ulk1 pathway increased lc3ii/lc3i, p62 protein expression
curcumin	Gp120 induces BV2 in microglia	15 μ Mol/L *in vitro* for 12 h	Inhibition of PI3K/AKT/IKK/NF-κB pathway, inhibition LC3II, Atg5 expression
Tanshinone IIA	Hypoxia model in primary rat cardiac myocytes	5μ Mol/L *in vitro* for 24 h	Activation of mTOR pathway, inhibition of lc3ii, Beclin1, p62 expression, lysosomal dysfunction

## Discussion

Autophagy is a highly complex physiological process that plays a crucial role in the defense of the body against diseases. Additionally, the occurrence and development of multiple oral disorders are associated with abnormal autophagy. Therefore, the intervention of autophagic activity opens new avenues for the prevention and treatment of diseases. Autophagy has a dual impact on oral diseases. First, it suppresses lesions at an early stage by eliminating damaged organelles and proteins, alleviating cell damage, and ensuring metabolic stability. Besides, once the lesion has formed, autophagy improves the survival rate of cells under a stressful environment, thereby playing a disease-promoting role. The effect of autophagy on the occurrence and development of oral diseases is mainly expressed through autophagy-related genes. Besides, the autophagy pathway has been found to cure oral diseases and has become the focus of much research as a result. Therefore, exploring the mechanism of autophagy and elucidating the relationship between the signaling tracks of autophagy and oral diseases may lead to new developments in treating oral conditions. Autophagy plays an essential role in the defense against microbial infections by clearing pathogens and modulating innate and acquired immune responses. Thus, in-depth studies of the relationship between autophagy and microbial infection are essential for exploring effective therapeutic avenues for infectious diseases. In recent years, some progress has been made determining the molecular mechanism of cell autophagy. However, many issues, such as the signal transduction pathway of autophagy or the origin of autophagy, are still unclear. Therefore, more attention should be paid to these problems in future studies. Additionally, natural substances are now more widely used in the clinical treatment of diseases. Plants produce a variety of natural compounds that cope with various environmental pressures. Many of these chemicals have pharmacological and clinical characteristics that constitute a library of natural small molecules and macromolecules for the discovery of drugs. A focus of future research will be to isolate sugar-binding metabolites from traditional medicinal plants and study their effects on autophagy, to discover new mechanisms of phytochemicals in the treatment of diseases.
